# Demographic profile and utilization statistics of a Canadian inpatient palliative care unit within a tertiary care setting

**DOI:** 10.3747/co.v16i1.172

**Published:** 2009-01

**Authors:** J. Napolskikh, D. Selby, M. Bennett, E. Chow, K. Harris, E. Sinclair, J. Myers

**Affiliations:** * Radiation Oncology, Odette Cancer Centre, Toronto, ON; † Palliative Care Consult Team, Sunnybrook Health Sciences Centre, Toronto, ON; ‡ Long Term Care, Sunnybrook Health Sciences Centre, Toronto, ON

**Keywords:** Palliative care, palliative care unit, health care services, length of stay, waiting time, service utilization

## Abstract

**Background:**

Canadian data describing inpatient palliative care unit (pcu) utilization are scarce. In the present study, we performed a quality assessment of a 24-bed short-term pcu with a 3-months-or-less life expectancy policy in a tertiary care setting.

**Methods:**

Using a retrospective chart review, we explored wait time (wt) for admission (May 2005 to April 2006), length of stay [los (February 2005 to January 2006)], and patient demographics.

**Results:**

The wt data showed 508 referrals, with 242 resulting in admissions (92% malignant diagnoses) and 266 not (82% malignant). The most common malignancies in both groups were gastrointestinal, lung, and genitourinary. Median wt for admitted patients was 6 days, varying with referral source, such as the same hospital, home, or another hospital (6, 4, and 8.5 days respectively). Most admissions (93%) occurred in 21 or fewer days. Patient death (52%), admission to another pcu (25%), and declined offer (10%) were common reasons for no admission. Median los for 219 admitted patients was 19 days (range: 0–249 days). Most patients (94%) died in the pcu; a minority were discharged.

**Conclusions:**

Many patients requiring pcu services are admitted within a few days of referral, especially patients with the least available support: those at home. However, half of the non-admitted patients die while waiting—a potential area for improvement. The los for admitted patients complied with the 3-month “expected lifespan” pcu policy. Results are significant, because ensuring quality of life for palliative care patients includes timely pcu access and sufficient los to address end-of-life needs.

## 1. INTRODUCTION

In the context of end-of-life care, palliative care specializes in symptom management and the provision of services to “relieve suffering and improve the quality of living and dying” 1,2. Although many patients state that they would prefer to die in the comfort of their own home with the support of home hospice 3, others prefer inpatient facilities such as a palliative care unit (pcu). The aim of a pcu is to provide holistic care by multidisciplinary teams including doctors, nurses, physiotherapists, occupational therapists, social workers, clergy, pharmacists, and volunteers 1,4. To be able to access this type of pcu care in Canadian hospitals, a physician referral is required—although some freestanding hospices can be accessed by client self-referral.

The modern hospice movement was founded in 1967 in England by Dame Cecily Saunders; the field of Canadian palliative care was established by Balfour Mount in 1974 5–7. Thus, palliation is still a rather new concept in patient care, and pcus are also in their early stages and in the process of reaching their “steady state.”

Currently, published Canadian data addressing pcu utilization and the demographics of the population accessing services in these units are scarce. A greater body of data is necessary to understand current approaches, to identify best practices, and subsequently to make evidence-based decisions in palliative care. Information concerning certain service aspects—such as waiting time (wt) for admission after referral, length of stay (los) for admitted patients, reasons that referrals do not result in admission for some patients, and demographic characteristics of the population requesting pcu services—is important for evaluating access to pcu services. Guidelines for an optimal los are also lacking, resulting in variation in pcu philosophy, such as “2-week,” “3-month,” and “6-month” units. Jenkins *et al.* reported a 21-day median los in an Edmonton pcu whose anticipated los was set at 2 months 4. Currently, Canadian studies have reported few wt data for pcu services, leaving a novel area for investigation. Furthermore, in the light of recent evaluations concerning waits for health services across Canada, information related to wt for pcu services is pertinent.

In the present descriptive study, we set out to perform a quality assessment of a 24-bed pcu at the Sunnybrook Health Sciences Centre, a tertiary care hospital affiliated with a major cancer centre in Toronto, Canada. Like many other pcus in Canadian hospitals and unlike acute pcus^7^, the Sunnybrook facility is designated for terminal care of patients with an anticipated prognosis of 3 months or less. Exploration included wt for admission, los, and patient demographics.

## 2. PATIENTS AND METHODS

We retrospectively reviewed patient charts and admission records. For the wt concept, we selected a 1-year span from May 1, 2005, to April 30, 2006. This range provided the most complete dataset. Similarly, a span from February 1, 2005, to January 31, 2006, was chosen for the los data. The demographic data collected were age, sex, primary diagnosis, and location at the time of referral. Dates of referral and admission or the reason that an admission did not take place were collected to explore issues pertaining to access to the facility. The overall wt was calculated, as were separate wts for patients coming from home, from within the same hospital, and from other hospitals. The wt calculations used the day that the referral request was received by the pcu and the day that admission was granted. Median los was calculated using the date that a patient received admission and the date that he or she died or was discharged. Reasons for patient discharge were noted.

## 3. RESULTS

Of the total 508 referrals made to the pcu during the study period, 242 patients (48%) were admitted; the remaining 266 patients (52%) did not receive admission.

### 3.1 Referrals Resulting in Admission

**FIGURE 1:**
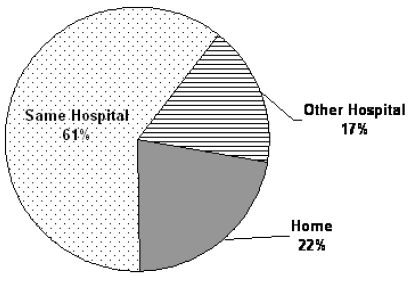
Referral sources for patients whose referrals eventually resulted in admission (*n* = 242).

Of the 242 admitted patients, 48% were men. Median age was 76 years (range: 39–97 years). Most of the patients (*n* = 223, 92%) had a malignant primary diagnosis. The most prevalent malignancies were gastrointestinal (colorectal, stomach, and esophageal) at 19%, lung at 19%, genitourinary (bladder, kidney, and prostate) at 13%, hematologic at 10%, and breast at 9%. Of the 8% of patients with non-malignant diagnoses, the most common was cardiovascular disease at 47% (*n* = 9). Other non-malignant diagnoses were hematologic, hepatic, renal, and neurologic disorders at 2 cases each. Sources of patient referral (Figure 1) were from within the same hospital (61%), from home (22%), and from other hospitals in the province of Ontario (17%). TABLE I fully outlines the demographic information.

**FIGURE 2:**
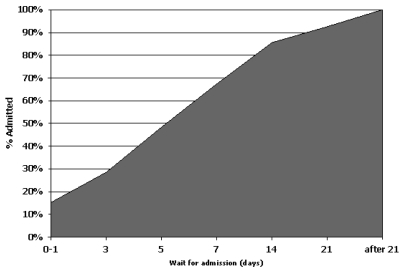
Waiting time distribution [*n* = 242 (non-admitted patients excluded)].

The median wait time between referral and admission was 6 days, ranging from same-day admission to admission at 306 days. Median wt varied according to the source of the referral: 4 days from home, 6 days for patients referred from Sunnybrook Health Sciences Centre, and 8.5 days for referrals from another hospital. Patients admitted within 24 hours of referral constituted 15% of the sample. Almost half the patients (*n* = 117, 48%) were admitted within 5 days, and 93% (*n* = 224) were in the pcu within 21 days (Figure 2).

A small number (*n* = 11) of charts indicated that the patient or the patient’s family refused the first offer, but accepted an offer at a later date and received admission. The dates of the initial offers were not available. However, the dates of the actual admissions were available, and although the resulting wt appeared perhaps unusually long for 2 patients, those records were not excluded from the median wt calculations to avoid potential bias in the results. Because the rest of such first-offer refusals (*n* = 9) did not result in longer wts and because some of the longest wts did not indicate a reason for the length of the wait, our consensus was that notes of a first offer refusal in patient records were not sufficient for exclusion of such records.

### 3.2 Referrals Not Resulting in Admission

**FIGURE 3:**
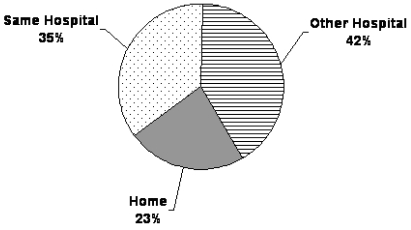
Referral sources for patients whose referrals did not result in admission (*n* = 266).

**FIGURE 4:**
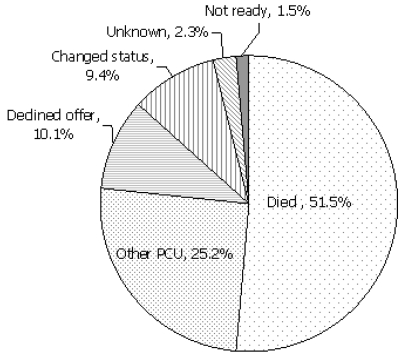
Reasons for no admission after referral (*n* = 266). pcu = palliative care unit.

The no-admission patient pool came from the same cohort as the patients who received admission, and this group was similar to the admitted group in age (median: 73 years; range: 25–102 years) and sex (50% men). The cancer diagnoses in the non-admitted patient group were similar in distribution to those in the admitted group (lung, 22%; gastrointestinal, 17%; genitourinary, 13%; gynecologic, 10%). Cardiovascular disease contributed 44% (*n* = 17) of the non-malignant diagnoses, and neurologic disorders accounted for 33% (*n* = 13). TABLE II and Figure 3 indicate the demographic profile of this patient group. Figure 4 shows the reasons that admissions never took place, including patient death (*n* = 137, 52%), admission of the patient to another pcu (*n* = 67, 25%), a choice to decline the offer (*n* = 27, 10%), or a change in patient status [for example, deciding to postpone the pcu referral (*n* = 25, 9%)]. Of the patients who died waiting (*n* = 137), 77% (*n* = 106) had a cancer diagnosis, and 19% (*n* = 26) had a non-malignant diagnosis. In 4% (*n* = 5), the diagnosis was unknown.

### 3.3 Length of Stay

Between February 1, 2005, and January 31, 2006, 219 patients were admitted to the pcu. As might be expected, the demographic profile of this group was similar to that of the group of admitted patients described earlier. TABLE III provides detailed demographic information.

**FIGURE 5:**
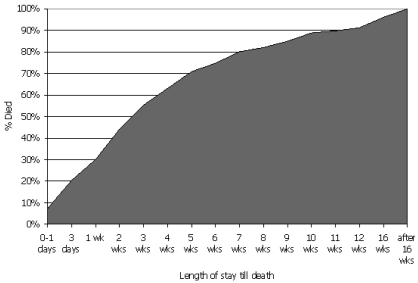
Length of stay before death [*n* = 219 (discharged patients excluded)].

The median los in the pcu was 19 days, ranging between 0 (death on the day of admission) and 249 days. Most patients (*n* = 206, 94%) died in the pcu; a small number (*n* = 13, 6%) were discharged. Patients dying in the pcu had a median stay of 19 days, ranging from 0 to 249 days. Interestingly, 7% (*n* = 15) of the admitted patients died in the first 24 hours. Almost one third (*n* = 62, 30%) died in the first week, and two thirds (*n* = 130, 63%) died within 4 weeks. By 3 months (12 weeks), 91% (*n* = 188) of the patients had died. Figure 5 shows the distribution of los before death. The 13 patients who were discharged from the pcu had a median stay of 77 days, ranging from 13 to 232 days. The reasons for discharge included transfer to a preferred pcu location (*n* = 1), a switch to more aggressive treatment (*n* = 1), and extended survival or an improvement in condition (*n* = 11, 5%). Of these latter 11 patients, only 1 was discharged home; the rest were discharged to other long-term care facilities. Data on subsequent outcome of the discharged patients were unavailable.

## 4. DISCUSSION AND CONCLUSIONS

Current statistics indicate that 1 in 4 Canadians will die of cancer and that approximately half of newly diagnosed patients eventually die of their disease^8^. Diagnosis and treatment of cancer often brings numerous symptoms, including pain in 80% of patients^9^. Symptoms have a tendency to increase in both number and severity with approaching death 10,11, making palliative care with the option of inpatient pcu admission a critical service for patients in the terminal phase of their illness. Most patients referred to our pcu (both admitted and not admitted) had a malignant diagnosis, which accords with other available Canadian studies^4^,^12^. In our pcu, the most common cancers were lung and gastrointestinal, followed by genitourinary and then hematologic and gynecologic cancers. Similarly, Jenkins *et al.* and Bruera *et al.* both reported that gastrointestinal and lung cancers were the most prevalent diagnoses in admissions to pcu, followed by breast, genitourinary, hematologic, and gynecologic malignancies 4,12.

Duration of wait for admission to a pcu after physician referral is an area of Canadian health care research that has not been previously addressed. A study of an acute 12-bed pcu (anticipated stay of less than 2 weeks) by Zimmermann *et al.* found a wt of 0–3 days 7, although patient turnover in this setting would be expected to be faster than that in a facility designated for longer stays and terminal care.

Our pcu grants admissions in the order of referral receipt, with the exception of patients coming from home, who receive higher priority. Median wt was consistent with the assigned priority: it was considerably shorter (4 days) for patients coming from home than for inpatients (6 and 8.5 days for same-institution and outside-institution referrals respectively). Bruera *et al.* found that their patient sample consisted mostly of elderly individuals living alone; however, even when the main caregivers were identified, 58% were unable to provide sufficient care at home because of their own health issues^12^. Although we did not specifically collect this type of information, similar situations may have been relevant to our patients, because their median age ranged from 73 to 77 years. Evidently, pcu care is a helpful alternative to dying at home, and in fact, Bruera *et al.* found that as many as 90% of 125 surveyed patients admitted to a pcu did not want to die at home^12^.

Two thirds of the referred patients who received admission waited 1 week or less, and most of the admissions (93%) occurred within 3 weeks. However, just over half the referrals that did not result in admission were unsuccessful because of patient death. Our study also found that many patients died shortly after admission. Among admitted patients, 7% died in the pcu in the first 24 hours after admission; by 1 week, 30% had died. This finding suggests that referral for pcu care may occur too late, possibly in part because of inadequate awareness about palliative services. Similar conclusions have been made in a number of studies addressing barriers to palliative care access^13^–^15^, and programs such as Living Lessons have been set up to increase public awareness of palliative care^16^. Schockett *et al.* found that late referral to end-of-life care, such as to outpatient hospice in the United States, was positively associated with lower patient and family satisfaction with care 14. This preliminary indication of reduced benefit, which may also apply to palliative care in Canada, supports the preparation programs mentioned earlier. Furthermore, because half of referred patients who did not receive admission died while waiting, expanding the number of beds in the 24-bed unit and partnering with an acute pcu may be practical solutions. Such outcomes may be of interest to Canadian hospitals and cancer centres currently considering opening a pcu.

The median los in our study was 19 days, which is consistent with other available studies. A number of U.S. studies looking at outpatient hospice utilization have examined the duration of end-of-life care, finding that the median generally ranges between 3 weeks and 1 month^13^,^15^,^17^–^19^. In a Canadian study from Edmonton, Alberta, Jenkins *et al.* retrospectively examined characteristics of an inpatient pcu with an anticipated los of 2 months and reported that, in a sample of 106, the median length of stay was 21 days, ranging between 0 and more than 200 days^4^. The authors suggested that patients may be able to manage their own care until the very final stages of their illness before requiring institutionalization^4^. In comparison, the mean los was 11 days at an acute pcu (designed predominately for acute symptom management) in Toronto, where the maximum los is set at 2 weeks^7^

In our pcu, los complied with the 3-month “expected lifespan” policy, with 91% (*n* = 188) of deaths occurring within 12 weeks of admission. However, 9% occurred after 3 months. Inability of family members to provide adequate support at home, lack of home care in the patient’s geographic location, and complexity of care required are all factors that may account for longer durations of stay. Such factors are also reflected in findings from Bruera *et al.,* who wrote that “likelihood for discharge [as determined by their palliative care team] was poor in 82% of cases, even if adequate symptom control had been achieved,” especially as a result of low family support and lack of appropriate health care services^12^.

Eleven patients (5%) were discharged because of symptom stabilization and longer-than-expected survival, and 1 patient switched to active treatment. This outcome is analogous to that in the Edmonton study by Jenkins *et al.,* in which 5% of patients were also transferred to longer term palliative care, and 2% moved to active treatment^4^.

Current literature has suggested the need for careful evaluation of all aspects of palliative care^20^. The results of our descriptive study are significant for patient care, because timely access to end-of-life health care is important for patients in the terminal phase of their illness, especially for those with little support at home. Additionally, a pcu stay that allows sufficient time to address symptoms and well-being in a holistic manner is imperative for provision of quality pcu care. To further address the need for evaluation of pcu services, future studies approaching patients and their families to assess their satisfaction with pcu services are warranted.

## Figures and Tables

**TABLE I:** Demographic information for the referred and admitted patients

Total referrals (*n*)	242
Age (years)	
Median (range)	76 (39–97)
Sex [% (*n*)]	
Men	48 (117)
Malignant diagnosis [% (*n*)]	92 (223)
Gastrointestinal	19 (42)
Lung	19 (43)
Genitourinary	13 (29)
Hematologic	10 (23)
Breast	9 (19)
Other	30 (67)
Non-malignant diagnosis [% (*n*)]	8 (19)
Cardiovascular disease	47 (9)
Hematologic disease	10.5 (2)
Liver disease	10.5 (2)
Neurologic disease	10.5 (2)
Renal disease	10.5 (2)
hiv	5.5 (1)
Unknown	5.5 (1)
Referral from home [% (*n*)]	22 (53)
Wait time [days; median (range)]	4 (1–306)
Referral from same hospital [% (*n*)]	61 (147)
Wait time [days; median (range)]	6 (0–289)
Referral from other hospital [% (*n*)]	17 (42)
Wait time [days; median (range)]	8.5 (1–98)
Overall wait time [days; median (range)]	6 (0–306)

**TABLE II:** Demographic information for patients referred, but not admitted

Total referrals (*n*)	266
Age (years)	
Median (range)	73 (25–102)
Sex [% (*n*)]	
Men	50 (132)
Malignant diagnosis [% (*n*)]	82 (217)
Lung	22 (47)
Gastrointestinal	17 (36)
Genitourinary	13 (28)
Gynecologic	10 (22)
Breast	8 (18)
Other	30 (66)
Non-malignant diagnosis [% (*n*)]	15 (39)
Cardiovascular disease	44 (17)
Neurologic disease	33 (13)
Liver disease	8 (3)
Renal disease	5 (2)
Hematologic disease	2.6 (1)
hiv	2.6 (1)
Lung disease	2.6 (1)
Spinal injury	2.6 (1)
Unknown diagnosis [% (*n*)]	3 (10)
Referral from home [% (*n*)]	23 (62)
Referral from same hospital [% (*n*)]	35 (94)
Referral from other hospital [% (*n*)]	42 (110)

**TABLE III:** Demographic information for the length-of-stay patient group

Total admissions *(n*)	219
Age (years)	
Median (range)	77 (39–97)
Sex	
Men [% (*n*)]	45 (99)
Malignant diagnosis [% (*n*)]	91 (199)
Gastrointestinal	20 (39)
Lung	14 (28)
Genitourinary	13 (26)
Hematologic	11.5 (23)
Breast	8 (16)
Other	33.5 (67)
Non-malignant diagnosis [% (*n*)]	9 (20)
Cardiovascular disease	45 (9)
Neurologic disease	15 (3)
Renal disease	15 (3)
Liver disease	10 (2)
Hematologic disease	10 (2)
hiv	5 (1)
Died [% (*n*)]	94 (206)
Length of stay [days; median (range)]	19 (0–249)
Discharged [% (*n*)]	6 (13)
Length of stay [days; median (range)]	77 (12–232)
